# Proinflammatory and cytotoxic response to nanoparticles in precision-cut lung slices

**DOI:** 10.3762/bjnano.5.253

**Published:** 2014-12-18

**Authors:** Stephanie Hirn, Nadine Haberl, Kateryna Loza, Matthias Epple, Wolfgang G Kreyling, Barbara Rothen-Rutishauser, Markus Rehberg, Fritz Krombach

**Affiliations:** 1 Walter Brendel Centre of Experimental Medicine, Ludwig-Maximilians-Universität München, Marchioninistr. 15, 81377 Munich, Germany; 2 Inorganic Chemistry and Center of Nanointegration Duisburg-Essen (CeNIDE), University of Duisburg-Essen, Universitätsstr. 5-7, 45117 Essen, Germany; 3 Institute of Epidemiology 2, Helmholtz Center Munich, Ingolstädter Landstr. 1, 85764 Neuherberg/Munich, Germany; 4 Adolphe Merkle Institute, Université de Fribourg, Route de l'ancienne Papeterie CP 209, 1723 Marly, Switzerland

**Keywords:** cytokines, cytotoxicity, ex vivo, lung slices, nanoparticles

## Abstract

Precision-cut lung slices (PCLS) are an established ex vivo alternative to in vivo experiments in pharmacotoxicology. The aim of this study was to evaluate the potential of PCLS as a tool in nanotoxicology studies. Silver (Ag-NPs) and zinc oxide (ZnO-NPs) nanoparticles as well as quartz particles were used because these materials have been previously shown in several in vitro and in vivo studies to induce a dose-dependent cytotoxic and inflammatory response. PCLS were exposed to three concentrations of 70 nm monodisperse polyvinylpyrrolidone (PVP)-coated Ag-NPs under submerged culture conditions in vitro. ZnO-NPs (NM110) served as ‘soluble’ and quartz particles (Min-U-Sil) as ‘non-soluble’ control particles. After 4 and 24 h, the cell viability and the release of proinflammatory cytokines was measured. In addition, multiphoton microscopy was employed to assess the localization of Ag-NPs in PCLS after 24 h of incubation. Exposure of PCLS to ZnO-NPs for 4 and 24 h resulted in a strong decrease in cell viability, while quartz particles had no cytotoxic effect. Moreover, only a slight cytotoxic response was detected by LDH release after incubation of PCLS with 20 or 30 µg/mL of Ag-NPs. Interestingly, none of the particles tested induced a proinflammatory response in PCLS. Finally, multiphoton microscopy revealed that the Ag-NP were predominantly localized at the cut surface and only to a much lower extent in the deeper layers of the PCLS. In summary, only ‘soluble’ ZnO-NPs elicited a strong cytotoxic response. Therefore, we suggest that the cytotoxic response in PCLS was caused by released Zn^2+^ ions rather than by the ZnO-NPs themselves. Moreover, Ag-NPs were predominantly localized at the cut surface of PCLS but not in deeper regions, indicating that the majority of the particles did not have the chance to interact with all cells present in the tissue slice. In conclusion, our findings suggest that PCLS may have some limitations when used for nanotoxicology studies. To strengthen this conclusion, however, other NP types and concentrations need to be tested in further studies.

## Introduction

Nanoparticles (NPs) are defined as materials with one dimension between 1–100 nm that occur naturally or anthropogenically. The class of synthetic NPs can be subdivided in incidental NPs that are generated as byproducts of combustion processes (e.g., in diesel exhaust) and engineered NPs (ENPs) that are specifically manufactured. The synthesis of ENPs allows one to control the properties of NPs such as size, shape, or chemical composition. As nanotechnology is a tremendously growing field with high commercial interest, ENPs are already used in several areas such as car industry, power engineering, optics, cosmetics, medicine, and pharmaceutics. This wide application range also raises questions about the safety of ENPs. Toxicological testing for most commercial ENPs is missing so far as there is no regulation for the fabrication and declaration of ENPs in consumer products. Assigned with the task to determine potential health risk effects of ENPs, nanotoxicology is a rapidly emerging research area [1]. There are several in vitro and in vivo models for testing the toxicity of nanoparticles. In vitro test systems, offering the advantages of easy handling and high-trough-put screening, can provide a first estimation of possible toxic effects. However, cell culture systems do not adequately reflect the real in vivo situation, whereas animal experiments are elaborative, cost-intensive, and the transferability from animal to human is often difficult. Furthermore, the aspect of the three “R’s” (replacement, refinement, and reduction of animal experiments) makes the development of in vitro or ex vivo alternatives a worthwhile objective [2–3]. Therefore, tissue slices can be an alternative to in vitro and in vivo studies [4]. Precision-cut lung slices (PCLS) are already used as an alternative ex vivo model in lung pharmacotoxicology [5]. As the lungs are also an important primary target organ for aerosolized ENPs, PCLS could have the potential to serve as an efficient tool in nanotoxicology. PCLS can be prepared from several species such as mouse or rat, but also from human lung tissue [6]. A major advantage of this tissue model is the retention of the lung architecture. The conservation of intact cells, including alveolar epithelial cells, alveolar macrophages, and dendritic cells, in their natural context has been shown in several studies by immunohistochemical staining [7–9]. Nevertheless, this in vitro system has also its limitations. There are obvious disadvantages in comparison to the in vivo situation such as no ventilation, no stretching, and no perfusion of the tissue. Moreover, dead cells are present at the slice surface due to the cutting process [10]. However, airway dynamics are preserved in PCLS which allows the monitoring of bronchoconstriction triggered by chemical stimuli [11–13].

So far, PCLS are already used in pharmacotoxicology studies to determine the response to allergens, chemicals, tobacco smoke, engine emissions, or oxidant air pollutions [5]. These studies mostly focused on the evaluation of airway constriction in PCLS caused by the tested substances [11,13–14]. Despite their established usage in pharmacotoxicology, there are only a few studies using PCLS in the field of nanotoxicology, yet. It was demonstrated that solid lipid NPs induced a cytotoxic response in PCLS, but only at very high concentrations (1 mg/mL and higher) [15–18]. Wohlleben et al. reported that a cobalt ferrite nanomaterial elicited a dose-dependent cytotoxicity as determined by the water-soluble tetrazolium salt (WST-1) assay and that incubation of PCLS with carbon nanotubes did not induce a cytotoxic response, whereas exposure to ZnO-NPs induced a strong cytotoxic response in PCLS [19–20].

The aim of the present study was to investigate whether PCLS have the potential to serve as a generally applicable effective tool in nanotoxicology. First, we determined the viability of PCLS up to 72 h by carrying out live/dead staining, lactate dehydrogenase (LDH) assay, and WST-1 assay. Second, we assessed the cytotoxic and proinflammatory response of PCLS to Ag-NPs through LDH assay, WST-1 assay, and ELISA assay for CXCL-1 and tumour necrosis factor-α (TNF-α) release. ZnO-NPs served as a soluble control and quartz particles as a non-soluble control, since both particle types have induced cytotoxic as well as inflammatory responses in in vitro as well as in vivo assays.

## Results

### Particle characterization

The mean hydrodynamic diameter of the Ag-NPs as determined by dynamic light scattering (DLS) was 116 nm and the polydispersity index (PDI) was below 0.3 for all measurements. The mean diameter of the metallic core as determined by scanning electron microscopy (SEM) was 65 nm. Furthermore, the mean hydrodynamic diameter of the Ag-NPs in culture medium (DMEM/F-12 Ham) as measured by DLS was 120 nm. The mean hydrodynamic diameter of the uncoated ZnO-NP (NM-110) in distilled water and culture medium was 413 nm and 271 nm and that of the quartz particles 1114 nm and 1843 nm, respectively.

### Viability of PCLS

PCLS with a thickness of 250 µm were prepared from Wistar rats and subsequently cultured in DMEM/F-12 Ham. Viability was determined at 4, 24, 48, and 72 h after incubation in medium by three different test assays: LDH-assay, live/dead staining, and WST-1 assay.

**Figure 1:**
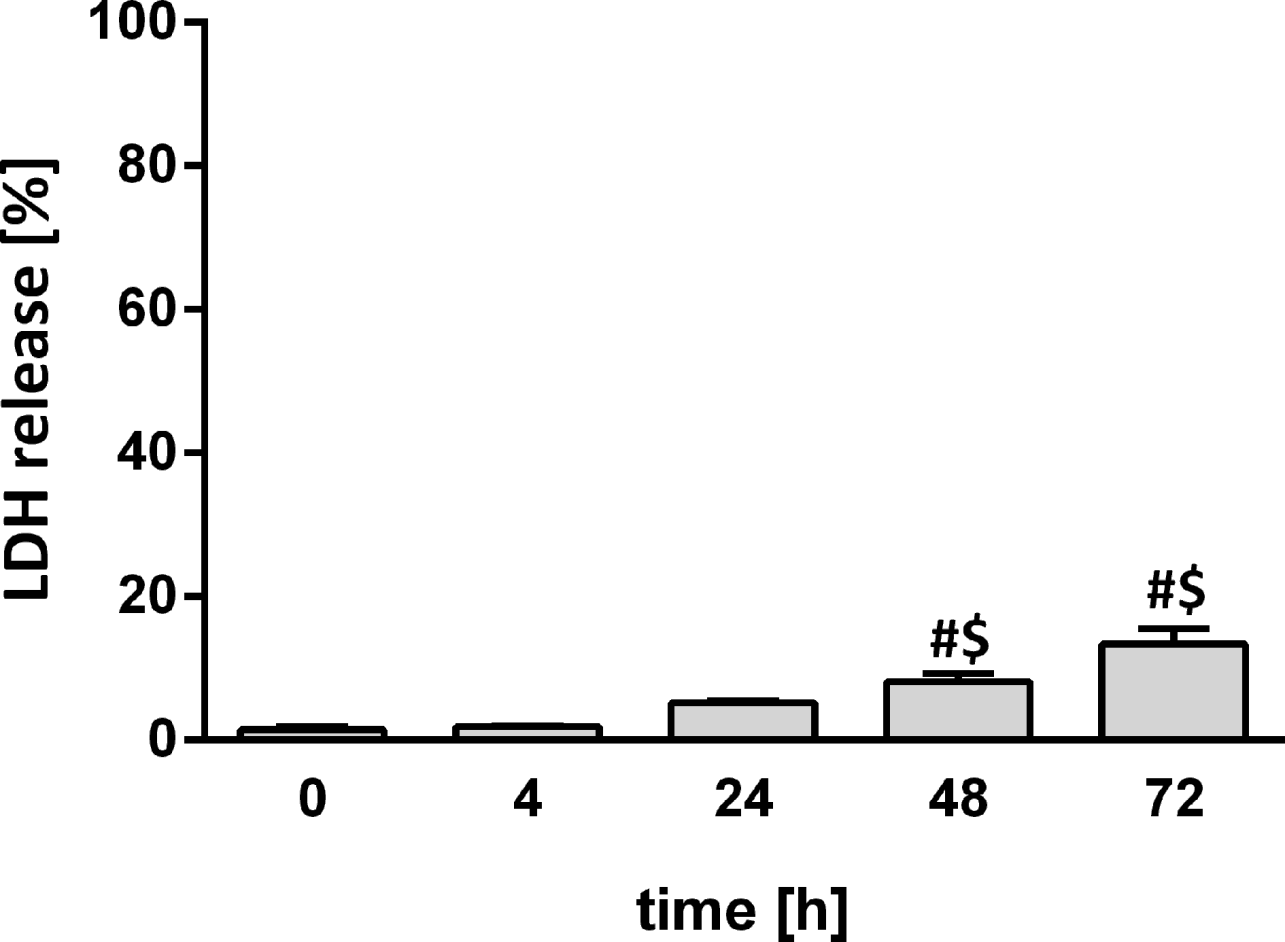
Cell viability as measured by LDH release in PCLS culture medium at 0, 4, 24, 48, and 72 h after preparation of PCLS. Results were calculated as percentage of the positive control (Triton X-100-lysed PCLS) (n = 5; ^#^*p* < 0.05 vs 0 h; ^$^*p* < 0.05 vs 4 h determined by ANOVA on ranks with post hoc Tukey test).

As LDH is present in the cytoplasm of cells, detection of LDH in the culture medium of PCLS indicates a loss of cell membrane integrity. Therefore, LDH release is a direct measure of a cytotoxic response or an indirect measure of cell viability. As displayed in Figure 1, the LDH release slightly increased over time, reaching 14% of total LDH after 72 h.

To corroborate the findings revealed by the LDH assay, we used live/dead staining as an additional viability test. While the ratio of spots (nuclei of dead cells)/volume (cytoplasm of living cells) did not change between 4 and 24 h of incubation, the ratio of spots/volume was doubled after 48 and 72 h indicating a slight decrease in viability over time (Table 1).

**Table 1:** Viability of PCLS determined by live/dead staining at 4, 24, 48, and 72 h after preparation of PCLS.

culture time	nuclei (diameter ≥ 4 µm)/volume (10^5^ µm^3^)

4 h	14 ± 1.4
24 h	13 ± 3.0
48 h	27 ± 1.2^a^
72 h	29 ± 1.9^a^

^a^*p* < 0.05 vs 4 and 24 h; determined by one-way repeated measures ANOVA with post hoc Tukey test.

**Figure 2:**
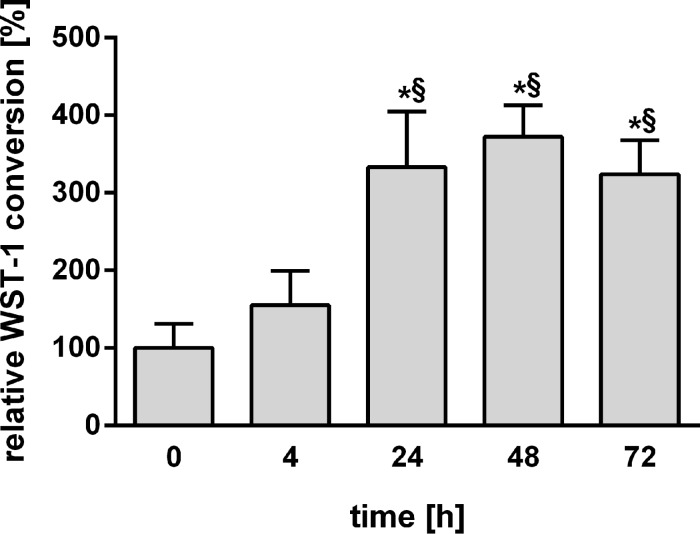
Viability of PCLS as determined by WST-1 conversion at 0, 4, 24, 48, and 72 h after preparation of PCLS (*n* = 5; *p < 0.05 vs 0 h; ^§^*p* < 0.05 vs 4 h determined by one-way ANOVA with post hoc Tukey test).

In a third viability test, the mitochondrial activity was measured by using WST-1 conversion. Interestingly, the mitochondrial activity increased after 24 h and persisted up to 72 h (Figure 2).

**Figure 3:**
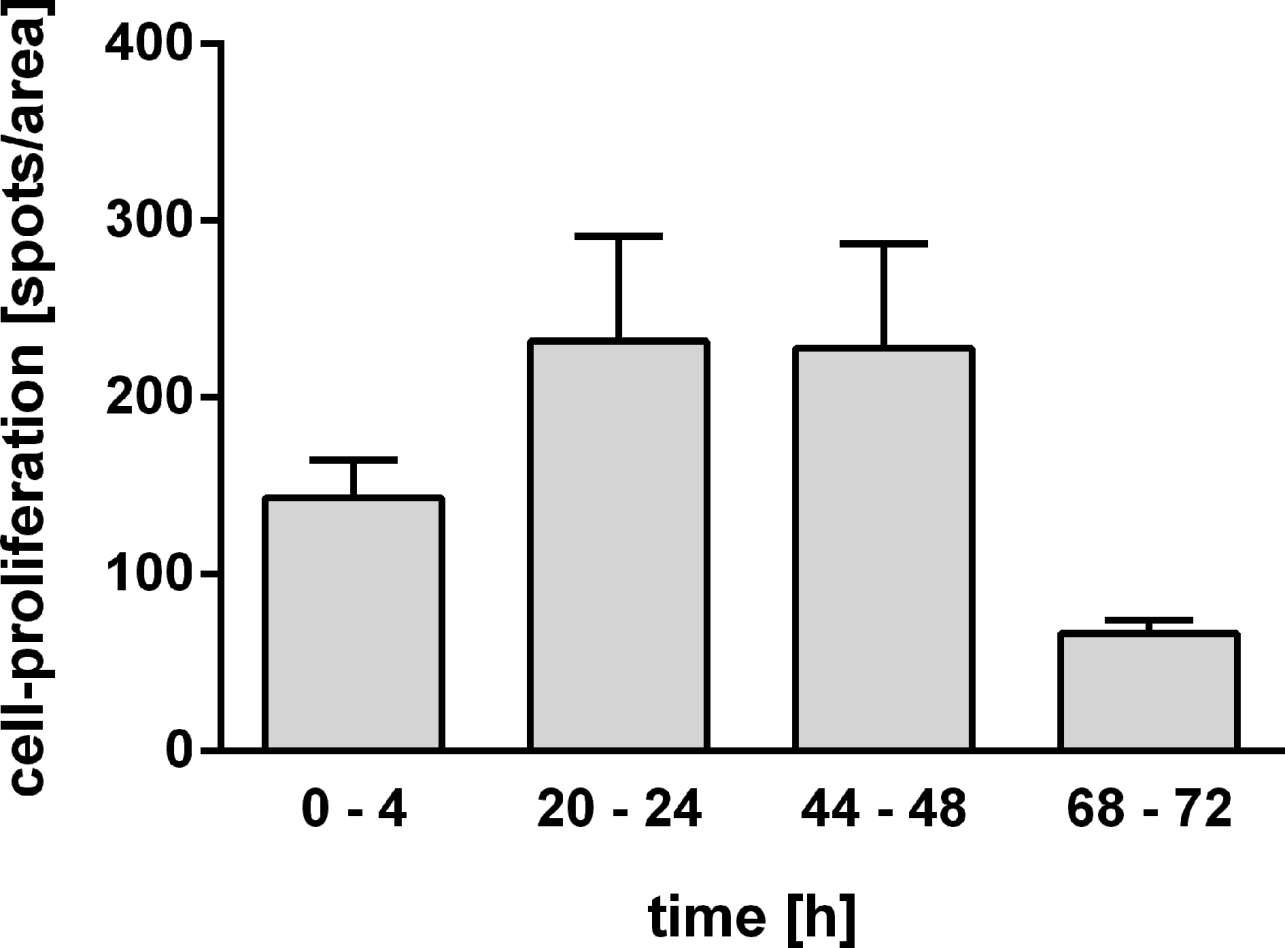
Cell proliferation in PCLS as determined by Click-iT^®^ EdU Alexa Fluor^®^ 488 Imaging Kit (Invitrogen) at four different time frames (*n* = 4 PCLS per time point, three different areas; no significant differences).

Since an increased WST-1 conversion could also indicate cell proliferation, we examined if there was enhanced cell proliferation by performing a proliferation assay (Click-iT^®^ EdU Alexa Fluor^®^ 488 Imaging Kit) in which the incorporation of 5-ethynyl-2′-deoxyuridine, a nucleoside analogue to thymidine, into replicated DNA can be visualized by confocal laser scanning microscopy. As shown in Figure 3, cell proliferation was not significantly altered as shown by this assay.

### Responses of PCLS after exposure to (nano)particles

#### Cytotoxic response

**Figure 4:**
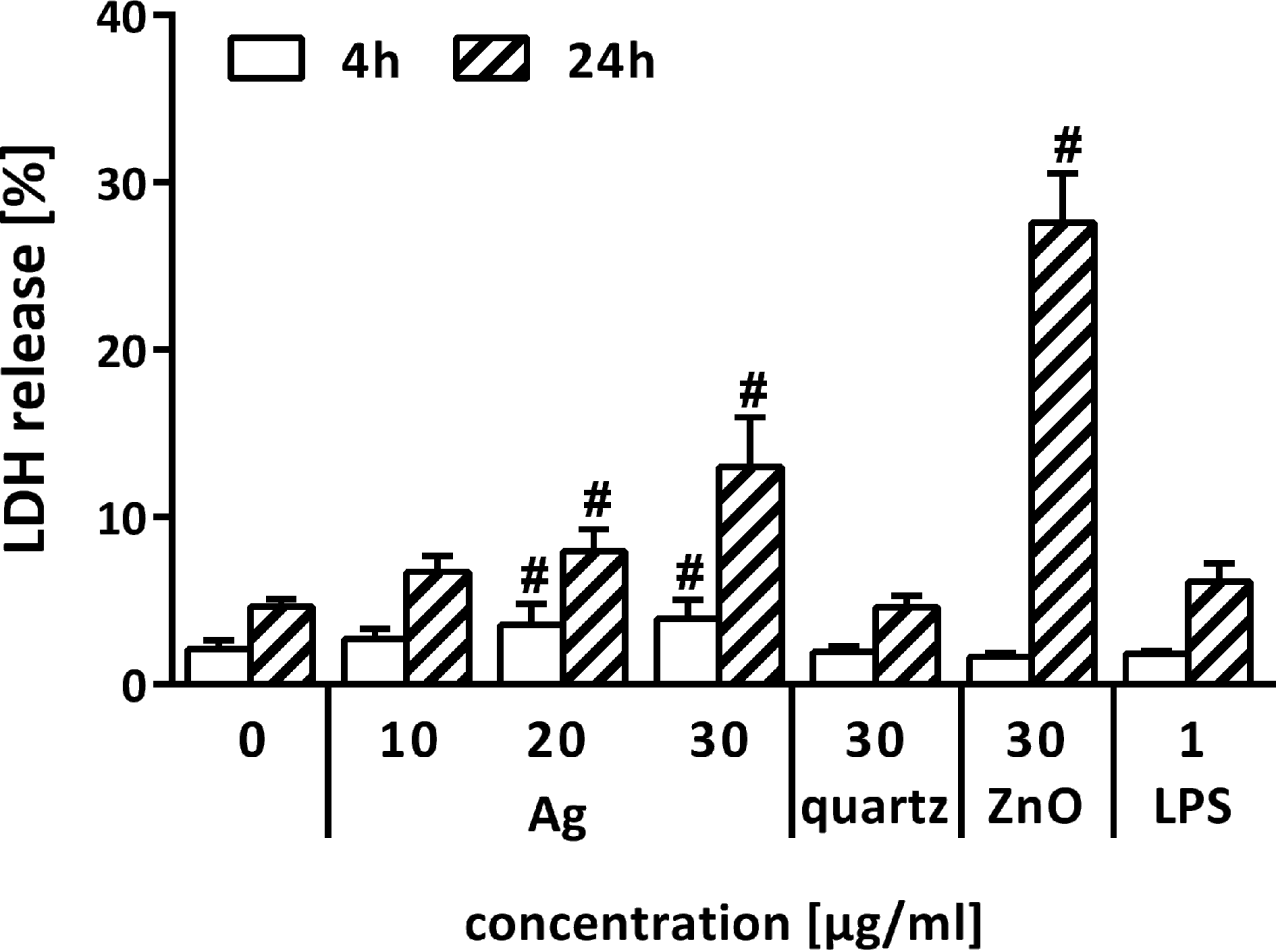
Cytotoxic response of PCLS as measured by LDH release after 4 and 24 h incubation. Results were calculated as percentage of the total LDH content, while Triton X-100 (Triton) lysed PCLS served as positive control with 100% LDH release (*n* = 5; ^#^*p* < 0.05 vs control determined by ANOVA on ranks with post hoc Student–Newman–Keuls test).

After 4 h of incubation with 20 and 30 µg/mL Ag-NPs, the LDH release was slightly increased to 4%, while exposure to ZnO-NPs and quartz particles did not result in increased LDH levels, indicating no cytotoxic response to these particles at this early time point. However, after incubation over 24 h, exposure to Ag-NPs caused a dose-dependent increase in LDH release of 8% and 13% after exposure to 20 and 30 µg/mL Ag-NPs, respectively. Moreover, 24 h of incubation with ZnO-NPs elicited a strong LDH release of 28%, while quartz particles did not induce a cytotoxic response (Figure 4).

**Figure 5:**
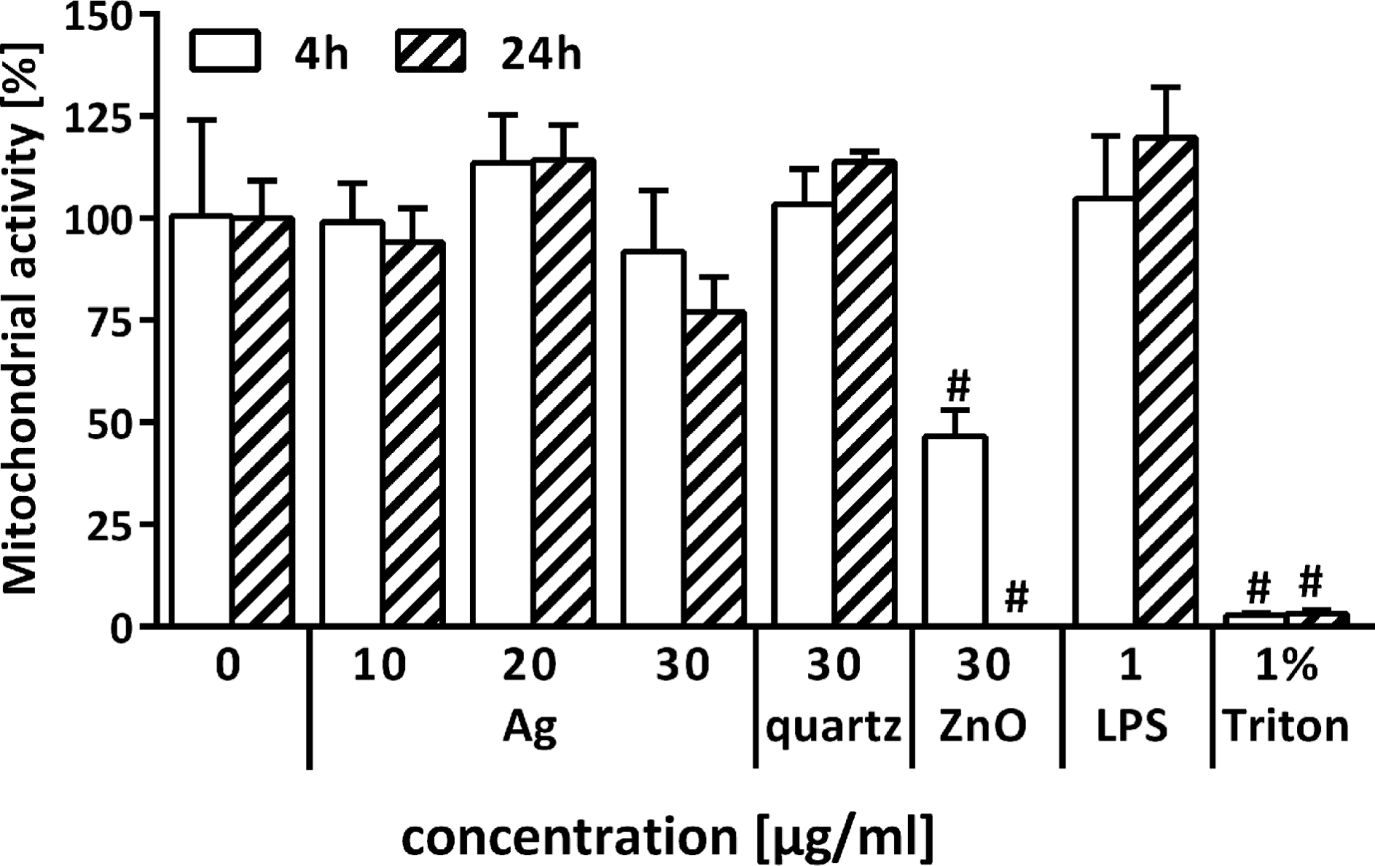
Cytotoxic response of PCLS as measured by WST-1 conversion after 4 and 24 h incubation (*n* = 5; ^#^*p* < 0.05 vs control determined by ANOVA on ranks with post hoc Student–Newman–Keuls test).

WST-1 conversion was measured as an additional cytotoxicity test. The mitochondrial activity did not change significantly after 4 and 24 h of incubation with Ag-NPs and quartz particles in comparison to controls. However, WST-1 conversion was decreased after 4 and 24 h of exposure to ZnO-NPs to 47% and to positive control level (PCLS treated with 1% Triton-X), respectively, again indicating a strong cytotoxic effect of these particles (Figure 5).

#### Proinflammatory response

**Figure 6:**
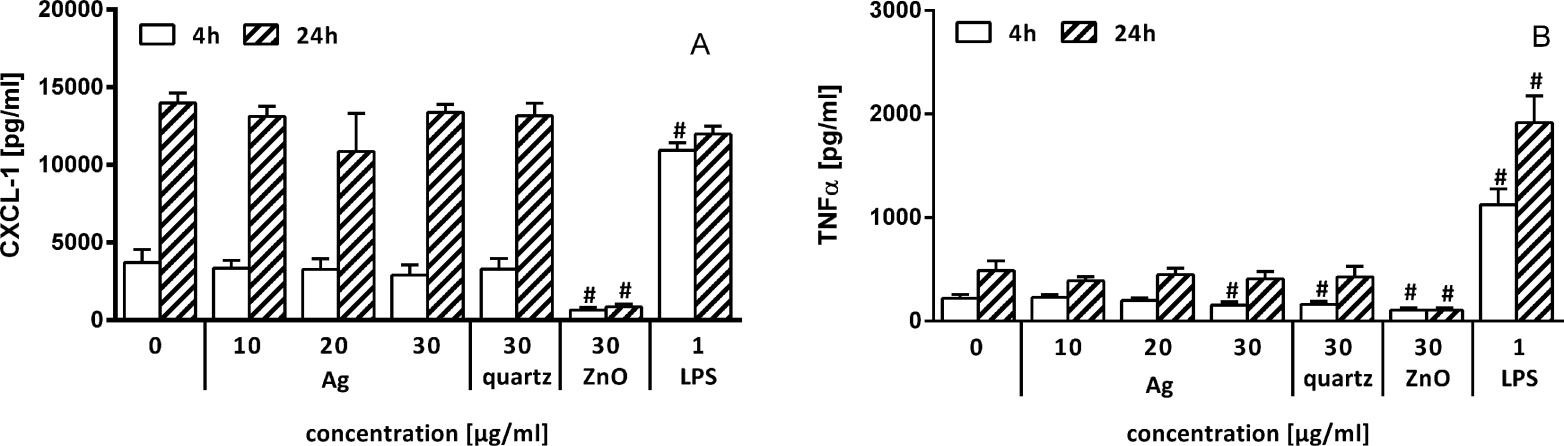
A: CXCL-1 levels measured in the PCLS culture medium after 4 and 24 h of incubation; B: TNF-α-levels measured in the PCLS culture medium after 4 and 24 h of incubation; *n* = 5; ^#^*p* < 0.05 vs control determined by ANOVA on ranks with post hoc Student–Newman–Keuls test.

The proinflammatory response in PCLS to NPs was quantified by measuring the levels of CXCL-1 and TNF-α in the PCLS culture medium through ELISA. The CXCL-1 release was not affected by incubation with Ag-NPs and quartz particles, while exposure to ZnO-NPs induced a slight decrease after 4 h of incubation. Exposure to lipopolysaccharide (LPS, positive control) elicited an increase in CXCL-1 levels after 4 h (Figure 6A). The release of TNF-α after 4 h was slightly decreased upon incubation with 30 µg/mL Ag-NPs, quartz particles, and ZnO-NPs, while LPS induced a strong increase in TNF-α levels. Whereas incubation of PCLS with Ag-NPs or quartz particles for 24 h did not induce a change in TNF-α levels, exposure to ZnO-NPs again resulted in a slight decrease in comparison to control levels (Figure 6B).

#### Localization of Ag-NPs in PCLS by using multiphoton microscopy

**Figure 7:**
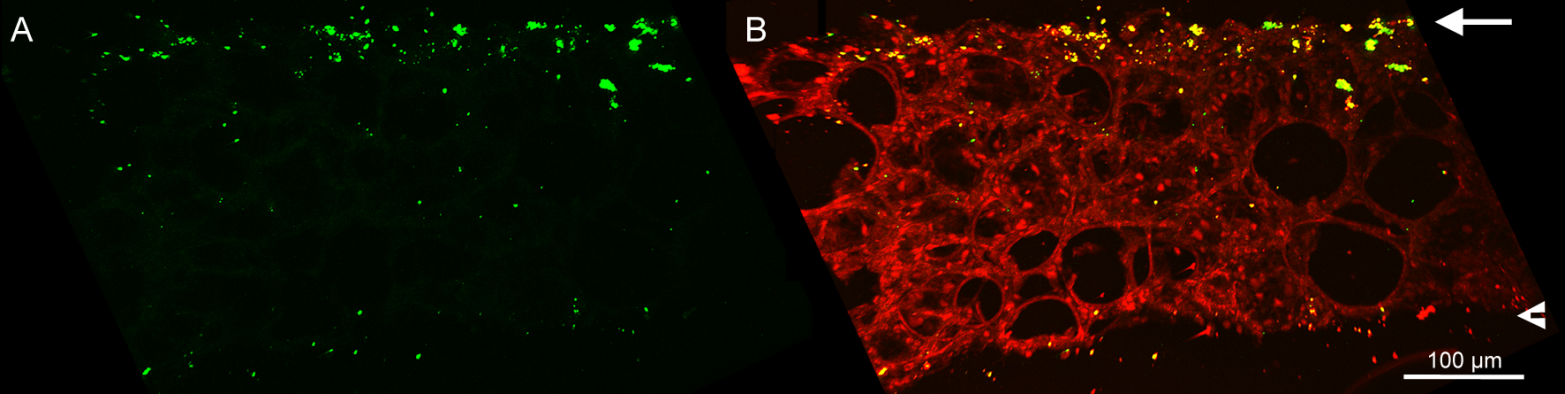
Multiphoton microscopy of Ag-NPs in the lung tissue. Image of an 80 µm median cryosection of PCLS incubated for 24 h with Ag-NPs (30 µg/mL). Red: signal of lung tissue; green: signal of Ag-NPs aggregates/agglomerates. A: green signal; B: merge of red and green signal. The arrows mark the upper cut surface, the arrow heads the lower cut surface.

To visualize the localization of NPs in PCLS after incubation, we employed multiphoton microscopy on PCLS exposed to Ag-NPs. By examining a median cross-cryosection of PCLS incubated with 30 µg/mL Ag-NPs for 24 h, a non-uniform distribution of Ag-NP aggregates/agglomerates was detected. As depicted in Figure 7, Ag-NP aggregates/agglomerates were predominantly localized on the upper cut surface of PCLS, a small amount on the lower cut surface and only a few Ag-NPs were found in inner tissue regions.

## Discussion

PCLS are already an established ex vivo alternative to in vitro and in vivo examinations in pharmacotoxicology. The aim of the present study was to evaluate the potential of PCLS as a tool in nanotoxicology studies.

First of all, we assessed the viability of PCLS over time by three different viability assays. The performance of more than just one viability test is essential as every test measures a different factor and, additionally, interference of particles with the test assay has been already described [21–22]. Taken together, a slight decrease in viability over time was detected which is also in line with results of previous studies with PCLS prepared from Wistar rat, guinea pig, and human lung tissue [10–11,23]. Also the viability of PCLS determined through live/dead staining slightly decreased over time, but PCLS were found to be still viable 72 h after preparation. Moreover, viability of PCLS was determined through WST-1 assay. The results of the WST-1 assay suggest that the viability of PCLS was improved 24 h after preparation. As WST-1 conversion is also an indicator for proliferation, we performed an additional proliferation assay that did not show significant differences in proliferation over time. The increase in mitochondrial activity after 24 h might be explained as recovery of the cells after the slicing procedure as it has been described already elsewhere [6,11]. Nevertheless, it has also been reported that the cutting process possibly induces the release of mitogenic factors that cause enhanced proliferation [24]. Taken together, the experimental findings of all three viability tests indicate that PCLS, in our hands, are viable for at least 72 h.

The aim of this study was to determine the suitability of PCLS as an alternative test system in nanotoxicology. Therefore, we assessed the cytotoxic and proinflammatory response of PCLS to Ag-NPs through LDH assay, WST-1 assay, and CXCL-1 and TNF-α ELISAs. The PVP-coated Ag-NPs used in this study have been already demonstrated to induce a cytotoxic and/or proinflammatory response in vivo and in vitro [25–26]. Moreover, ZnO-NPs served as a soluble control and quartz particles as a non-soluble control, since both particle types have been shown to elicit cytotoxic as well as inflammatory responses in both in vitro and in vivo assays [27–28].

Interestingly enough, the PVP-coated Ag-NPs used in this study only induced a slight cytotoxic, but not an inflammatory response in PCLS. However, these NPs induced cytotoxicity in human mesenchymal stem cells and peripheral blood mononuclear cells in vitro when incubated at a concentration of 25 µg/mL or higher [29–30]. Additionally, these NPs elicited a cytotoxic and inflammatory response in rat lungs 24 h after intratracheal instillation of 80 µL Ag-NPs at a concentration of 250 µg/mL [25]. Furthermore, quartz particles did not appear to cause a loss in viability or proinflammatory response, although a cytotoxic and/or inflammatory effect has been described after exposure to quartz particles in vitro as well as in vivo [28,31–33]. In particular, Beyerle et al. have demonstrated that quartz particles (the same as those in the present study) induced a loss in membrane integrity in vitro in murine alveolar epithelial cells and macrophages after 24 h of incubation [31]. Furthermore, cytotoxicity elicited by quartz particles has been shown in vivo by evaluation of bronchoalveolar lavage fluids and lung tissue of rats after intratracheal instillation [24,28]. In contrast, ZnO-NPs induced a strong cytotoxic effect after 4 and 24 h of incubation, which is in line with results of a previous report [20]. In this study, cytotoxicity was determined after a 24 h exposure of PCLS, also prepared from Wistar rats, to ZnO-NPs (the same as those in our study) or carbon nanotubes that induced either a marked or no cytotoxic response, respectively. Furthermore, a strong cytotoxic response after exposure with ZnO-NPs has been reported in several in vitro and in vivo studies [27,34–37]. However, the loss in viability after ZnO-NP exposure is presumed to be mainly induced by the release of Zn^2+^ ions [37]. The dissolution of NPs into toxic ions seems to be an important factor regarding the toxicity of the material [38–39]. Dissolution and therefore release of Zn^2+^ ions in the culture medium has been already described for the ZnO-NPs used in the present study [40]. Moreover, the dissolution (50 to 60% within 24 h) of ZnO-NPs in cell culture medium facilitates an interaction of Zn^2+^ ions with cells [27]. Ag-NPs were shown to dissolve after immersion in water in the presence of oxygen under the release of Ag^+^ ions [30,41]. However, their dissolution in biological environments is still poorly understood and will be influenced by the presence of biomolecules such as proteins [42–43]. Taken together, the viability of PCLS only decreased after exposure to ZnO-NPs, but not after exposure to quartz particles and only slightly after exposure to Ag-NPs.

Moreover, we determined the proinflammatory response of PCLS upon particle exposure. Surprisingly, none of the used materials induced a proinflammatory effect, although ZnO-NPs as well as quartz particles have been shown to induce inflammatory responses in the lung [27–28,34,36–37]. The low levels of CXCL-1 and TNF-α after exposure to ZnO-NP are probably due to the fact that most of the cells are already dead, as determined by the WST-1 assay. In summary, PCLS did not react with the release of proinflammatory cytokines upon exposure to the particles at the concentrations tested here.

The low cytotoxic response to Ag-NPs, the absent cytotoxic response to quartz particles, and also the non-existent inflammatory response to all particles are in contrast to studies using these particles in conventional in vitro and in vivo assays. Consequently, the question was whether and to what extent the particles interacted with the PCLS under submerged conditions. Therefore, we employed multiphoton microscopy to visualize the localization of Ag-NPs in and on PCLS after 24 h of incubation. Recently, our group has demonstrated that third harmonic generation (THG) microscopy, which is based on optical effects induced by specific inherent physical properties of a specimen, allows high-resolution label-free 3D visualization of cellular and tissue structures in intact muscle of living mice [44]. This technique allowed us to record images of Ag-NPs and lung tissue without additional labelling, since Ag-NPs exhibit strong two-photon-induced photoluminescence and enhanced THG signals through surface plasmon resonance [45–46]. As shown by multiphoton microscopy, the Ag-NPs were predominantly localized at the upper cut surface of PCLS as aggregates/agglomerates and only at a much lower extent in in deeper regions of the tissue slice. This indicates that the majority of the particles did not have the chance to interact with all cells present in the tissue slice since the Ag-NPs with their hydrodynamic diameter of 116 nm are too large for diffusional transport through the tissue. In contrast, the Zn^2+^ ions released by the ZnO-NPs are able to travel by diffusion, resulting in the strong cytotoxic response of PCLS we have measured in this study. These findings suggest that the interaction of non-soluble NPs with the majority of cells present in a given PCLS appears to be limited.

## Conclusion

Although a recently published study demonstrated that PCLS are able to detect different early effects of NP toxicity, it is still not clear whether PCLS possess an added value in detecting nanomaterial pulmonary toxicity [47]. Here we show that there was no cytotoxic response of PCLS to micron-sized quartz particles, only a slight cytotoxic response to PVP-coated Ag-NPs, but a strong cytotoxic response to uncoated ZnO-NPs. Moreover, none of the materials induced a proinflammatory effect in PCLS. Furthermore, we were able to show by multiphoton microscopy that the majority of Ag-NPs was localized on the cut surface of the PCLS and not able to translocate into inner regions of the tissue slice. Based on these findings, we conclude that PCLS may have some limitations when used in nanotoxicology studies. However, a definite conclusion can be only drawn after testing also other types and concentrations of NPs as well as using other more sensitive analysis methods to assess possible cell responses.

## Experimental

### Particles

PVP-coated Ag-NPs were prepared as described before [30,48]. Briefly, Ag-NPs were synthesized by reduction with glucose in the presence of PVP and dispersed in ultrapure degassed water. The silver concentration was determined by atomic absorption spectroscopy (AAS) with a detection limit of 1 μg·L^−1^. SEM was performed with a FEI Quanta 400 ESEM instrument in high vacuum without sputtering. Particle size distribution and PDI were measured by DLS with a Malvern Zetasizer Nano ZS (Malvern Instruments GmbH, Herrenberg, Germany).

The uncoated ZnO-NPs NM110 belonged to a set of representative manufactured nanomaterials provided and characterized by the European Commission Joint Research Centre (JRC, Ispra, Italy). Particle size distribution was determined by DLS. Shortly before incubation with PCLS, ZnO-NPs were dispersed following the Nanogenotox dispersion protocol [49].

The quartz particles (Min-U-Sil 5, crystalline silica, α-quartz) with a purity of 98% SiO_2_ were obtained from US Silica Company (Berkeley Springs, WV, USA). Immediately before incubation with PCLS, quartz particles were dispersed at a concentration of 1 mg/mL in distilled water. Subsequently, the suspension was vortexed for 1 min followed by ultrasonic bath for 1 min. This procedure was repeated three times.

### Animals

Female Wistar rats (Wistar RjHan:WI rats), 8–10 weeks of age (approx. 250 g body weight), were obtained from Janvier (Le Genest Saint Isle, France). The animals had at least one week for acclimatization and were housed in pairs in standard Makrolon plastic cages type IV on a 12 hour day/night circle. Rodent diet and water were provided ad libitum.

### Preparation of PCLS

Under isoflurane anaesthesia, rats were sacrificed by exsanguination through the abdominal aorta. Lungs were filled with 15 mL of a 1.5% low gelling temperature agarose solution in situ. After five minutes the lungs were extracted and cooled in an ice-cooled Earl’s Balanced Salt Solution for 10 min. Punched out cylinders (diameter 8 mm) of these lungs were cut into approximately 250 micrometre thin slices by using a Krumdieck tissue slicer (microtome; Alabama Research and Development, Munford, AL, USA). Always four PCLS per well (24-well plate) were cultured in Dulbecco’s modified Eagle’s medium/nutrient mixture F-12 Ham (DMEM/F-12 Ham) containing 100 units/mL penicillin and 100 µg/mL streptomycin. The 24-well plates were placed on a plate shaker in the incubator (5% CO_2_, 100% humidity, 37 °C). Prior to incubation, the PCLS were placed in a 24-well culture dish in the incubator for re-melting the agarose and washed for 1 h by changing the culture medium three times, in order to remove the agarose.

### Incubation of PCLS

For viability testing, PCLS were incubated with 500 µL DMEM/F-12 Ham for 4, 24, 48, and 72 h. For particle exposure experiments PCLS were exposed for 4 and 24 h to 500 µL DMEM/F-12 Ham either with PVP-Ag-NPs (10, 20, and 30 µg/mL), 30 µg/mL ZnO-NPs, or 30 µg/mL quartz particles. Therefore, after the 1 h washing procedure, culture medium used for washing was replaced by the particular suspension. Additionally, PCLS were incubated only with 500 µL DMEM/F-12 or with 500 µL DMEM/F-12 with 1 µg/mL LPS, which served as negative and positive (inflammatory) control. Furthermore, PCLS were lysed by adding 1% Triton X-100 which was used as positive (cytotoxic) control. All incubations were performed in duplicates.

### Determination of LDH release in PCLS culture medium

LDH release was determined in PCLS culture medium using the Cytotoxicity Detection Kit^PLUS^ (LDH) (Roche, Mannheim, Germany). Triton X-100 lysed PCLS were used as positive controls (100% LDH release). Results were calculated as percentage of the total LDH content. All tests were performed in duplicates.

### Live/dead staining

Viability of PCLS was determined after 4, 24, 48, and 72 h by using the LIVE/DEAD^®^ Viability/Cytotoxicity Kit for mammalian cells (Invitrogen, Karlsruhe, Germany). PCLS were incubated with 4 µM calcein acetoxymethyl (calcein AM) and 4 µM ethidium-homodimer-1 (EthD-1) for 45 min at room temperature. Live cells enzymatically convert calcein AM in fluorescent calcein, while EthD-1 enters dead cells through the damaged membrane, binds to their nuclei, and emits red fluorescence. Immediately after the staining procedure, PCLS were mounted in phosphate buffered saline (PBS) on glass slides and observed by using a Leica SP5 confocal laser-scanning microscope (cLSM, Leica Microsystems, Wetzlar, Germany) with a PlanFluotar objective (Leica; 20×; NA 0.5). The excitation wavelength was 488 nm and the emission was detected at 510–540 nm for calcein and 580–620 nm for EthD-1. Optical *z*-sections covering 30 µm depth of the PCLS (*z*-distance 1 µm) were recorded by using the same settings for all samples. Images were processed and analysed with Imaris software (Bitplane, Zürich Switzerland). For quantification of dead cells, the Imaris spotfinder algorithm was applied to automatically identify and count labelled cell nuclei in these 3D volumes and the volume of live cells was identified and calculated by using the surface tool. Similarly, as already described in Henjacovic et al. [8], viability was determined as ratio of nuclei of dead cells per volume of live cells (spots (diameter ≤ 4 µm)/volume (10^5^ µm^3^).

### WST-1 reduction in PCLS culture medium

The WST-1 assay (Roche Diagnostics, Mannheim, Germany) was used for spectrometric quantification of cellular viability. After removing cell culture medium, PCLS were incubated for 1 h with 500 µL freshly prepared WST-1 solution (manufacturer protocol) diluted 1:10 in DMEM/F-12 Ham. Absorbance was determined at a wavelength of 450 nm. Results are given in percentage of control. The relative WST-1 conversion was calculated as percentage of control (WST-1 conversion at 0 h). All tests were performed in duplicates.

### Cell proliferation assay

For determination of cell proliferation, the Click-iT^®^ EdU Alexa Fluor^®^ 488 Imaging Kit (Invitrogen, Karlsruhe, Germany) was used. PCLS were incubated for 4 h with 20 µM EdU (5-ethynyl-2′-deoxyuridine, a nucleoside analogue to thymidine) at 0, 20, 44, and 68 h after preparation. Subsequently, four PCLS per time point were fixed with buffered 4% paraformaldehyde (Microcos GmbH, Saaldorf-Surheim, Germany) at room temperature for 15 min. Then PCLS were washed twice in PBS supplemented with 3% bovine albumin serum (BSA) and stored at 4 °C until further processing. The EdU staining was conducted as described by the manufacturer. Briefly, after permeabilization (with PBS, supplemented with 3% BSA and 0.5% Triton X-100 (Sigma-Aldrich, Munich, Germany), PCLS were incubated with the EdU Click-iT® reaction cocktail followed by another washing step and finally mounted in PermaFluor (Thermo Scientific, Fremont, CA, USA) on a glass slide. Images were acquired with the cLSM (see details above). Excitation wavelength was 488 nm and emission was detected at 510–600 nm. Optical *z*-sections covering the complete depth of the PCLS (*z*-size 250 µm, *z*-distance 3 µm) were recorded by using the same settings for all samples. Images were processed and analyzed by using Imaris software (Bitplane, Zürich, Switzerland). For quantification of EdU positive cells, the Imaris spotfinder algorithm was applied to automatically identify and count labelled cell nuclei in three 3D-volumes (*xy*-size 500 µm, *z*-size 250 µm) from each PCLS sample.

### Quantification of TNF-α and CXCL-1 in the PCLS-culture medium by ELISA

The proinflammatory cytokines TNF-α and CXCL-1 were measured in PCLS supernatants by using commercially available enzyme-linked immunosorbent assay kits (ELISA, DuoSet, R&D, Wiesbaden-Nordenstadt, Germany) according to the specifications of the manufacturer. Absorbance was determined at a wave length of 450 nm (Tecan Infinite F200).

### Multiphoton microscopy

PCLS incubated with 30 µg/mL of Ag-NPs for 24 h were fixed in 70% ethanol until further processing. Before cryosectioning, PCLS were embedded in Tissue-Tek OCT compound (Miles, Inc. Diagnostic Division, Elkhart, IN) and frozen until hardening. Then 60 to 80 µm thick cross sections of PCLS were cut with a cryostat microtome (Microm HM560, Thermo scientific, Waltham, MA, USA), transferred on a glass slide, and coverslipped immediately.

Two-photon microscopy on PCLS sections incubated with Ag-NPs (30 µg/mL) was performed on a TriMScope (LaVision Biotec, Bielefeld, Germany) built around an Olympus BX 51 microscope (Olympus, Hamburg, Germany) and equipped with a tunable Ultra II Ti:sapphire laser (Coherent, Dieburg, Germany) and an optical parametric oscillator (Chameleon OPO; APE, Berlin, Germany), which is pumped by the Ti:sapphire laser. The OPO generated 1275 nm light with 640–700 mW output. The attenuated intensity at the sample was 170 mW. An Olympus XLUMPlanFl 20×/0.95W objective was used to acquire optical sections (*z*-size: 85 µm, *z*-distance: 1 µm, *xy*: 500 × 500 µm with 1035 × 1035 pixels and 400 lines per second).

The Ag-NP signal was (epi-)detected at 604–644 nm, THG signal was detected in the forward direction at (417–477 nm). 700 nm short pass filters blocked out excitation light. Light collection in forward direction was performed by an Olympus WI-UCD condenser, NA 0.8. Photomultiplier tubes were gallium arsenide phosphide detectors (Hamamatsu H7422-40). Images were processed and 3D rendered by using Imaris software (Bitplane, Zürich, Switzerland).

### Statistical analysis

Data in the figures are given as mean ± SEM (standard error of the mean). Statistical analysis was performed for normalized data by one-way ANOVA with post hoc Tukey test. Other data were analyzed by repeated measures ANOVA on ranks followed by a post hoc test as stated individually (Software: SigmaStat for Windows, Jandel Scientific, Erkrath, Germany). Differences were considered statistically significant at *p* < 0.05.

## References

[1] Maynard A D, Warheit D B, Philbert M A (2011). Toxicol Sci.

[2] Bach P H, Vickers A E M, Fisher R, Baumann A, Brittebo E, Carlile D J, Koster H J, Lake B G, Salmon F, Sawyer T W (1996). ATLA, Altern Lab Anim.

[3] Russell W M S, Burch R L (1959). The principles of humane experimental technique.

[4] Parrish A R, Gandolfi A J, Brendel K (1995). Life Sci.

[5] Morin J-P, Baste J-M, Gay A, Crochemore C, Corbiére C, Monteil C (2013). Xenobiotica.

[6] Liberati T A, Randle M R, Toth L A (2010). Expert Rev Mol Diagn.

[7] Davidovich N, Huang J, Margulies S S (2013). Am J Physiol: Lung Cell Mol Physiol.

[8] Henjakovic M, Sewald K, Switalla S, Kaiser D, Müller M, Veres T Z, Martin C, Uhlig S, Krug N, Braun A (2008). Toxicol Appl Pharmacol.

[9] Veres T Z, Voedisch S, Spies E, Tschernig T, Braun A (2011). Am J Pathol.

[10] Ressmeyer A R, Larsson A K, Vollmer E, Dahlén S E, Uhlig S, Martin C (2006). Eur Respir J.

[11] Martin C, Uhlig S, Ullrich V (1996). Eur Respir J.

[12] Martin C, Uhlig S, Ullrich V (2001). Am J Respir Cell Mol Biol.

[13] Schleputz M, Rieg A D, Seehase S, Spillner J, Perez-Bouza A, Braunschweig T, Schroeder T, Bernau M, Lambermont V, Schlumbohm C (2012). PLoS One.

[14] Henjakovic M, Martin C, Hoymann H G, Sewald K, Ressmeyer A R, Dassow C, Pohlmann G, Krug N, Uhlig S, Braun A (2008). Toxicol Sci.

[15] Nassimi M, Schleh C, Lauenstein H D, Hussein R, Hoymann H G, Koch W, Pohlmann G, Krug N, Sewald K, Rittinghausen S (2010). Eur J Pharm Biopharm.

[16] Nassimi M, Schleh C, Lauenstein H-D, Hussein R, Lübbers K, Pohlmann G, Switalla S, Sewald K, Müller M, Krug N (2009). Inhalation Toxicol.

[17] Neuhaus V, Schwarz K, Klee A, Seehase S, Forster C, Pfennig O, Jonigk D, Fieguth H G, Koch W, Warnecke G (2013). PLoS One.

[18] Paranjpe M, Neuhaus V, Finke J H, Richter C, Gothsch T, Kwade A, Büttgenbach S, Braun A, Müller-Goymann C C (2013). Inhalation Toxicol.

[19] Wohlleben W, Kolle S N, Hasenkamp L-C, Böser A, Vogel S, von Vacano B, van Ravenzwaay B, Landsiedel R (2011). J Phys: Conf Ser.

[20] Wohlleben W, Meier M W, Vogel S, Landsiedel R, Cox G, Hirth S, Tomović Z (2013). Nanoscale.

[21] Monteiro-Riviere N A, Inman A O, Zhang L W (2009). Toxicol Appl Pharmacol.

[22] Wörle-Knirsch J M, Pulskamp K, Krug H F (2006). Nano Lett.

[23] Wohlsen A, Martin C, Vollmer E, Branscheid D, Magnussen H, Becker W-M, Lepp U, Uhlig S (2003). Eur Respir J.

[24] Vallan C, Friis R R, Burri P H (1995). Exp Lung Res.

[25] Haberl N, Hirn S, Wenk A, Diendorf J, Epple M, Johnston B D, Krombach F, Kreyling W G, Schleh C (2013). Beilstein J Nanotechnol.

[26] Chernousova S, Epple M (2013). Angew Chem, Int Ed.

[27] Kermanizadeh A, Pojana G, Gaiser B K, Birkedal R, Bilanicova D, Wallin H, Jensen K A, Sellergren B, Hutchison G R, Marcomini A (2013). Nanotoxicology.

[28] Warheit D B, Webb T R, Colvin V L, Reed K L, Sayes C M (2007). Toxicol Sci.

[29] Greulich C, Diendorf J, Simon T, Eggeler G, Epple M, Köller M (2011). Acta Biomater.

[30] Kittler S, Greulich C, Diendorf J, Köller M, Epple M (2010). Chem Mater.

[31] Beyerle A, Schulz H, Kissel T, Stoeger T (2009). J Phys: Conf Ser.

[32] Cho W-S, Duffin R, Howie S E M, Scotton C J, Wallace W A H, Macnee W, Bradley M, Megson I L, Donaldson K (2011). Part Fibre Toxicol.

[33] Journeay W S, Suri S S, Moralez J G, Fenniri H, Singh B (2008). Small.

[34] Sayes C M, Reed K L, Warheit D B (2007). Toxicol Sci.

[35] Kermanizadeh A, Vranic S, Boland S, Moreau K, Baeza-Squiban A, Gaiser B K, Andrzejczuk L A, Stone V (2013). BMC Nephrol.

[36] Wilhelmi V, Fischer U, van Berlo D, Schulze-Osthoff K, Schins R P, Albrecht C (2012). Toxicol In Vitro.

[37] Xia T, Kovochich M, Liong M, Mädler L, Gilbert B, Shi H, Yeh J I, Zink J I, Nel A E (2008). ACS Nano.

[38] Inoue K, Branigan D, Xiong Z-G (2010). J Biol Chem.

[39] Misra S K, Dybowska A, Berhanu D, Luoma S N, Valsami-Jones E (2012). Sci Total Environ.

[40] Singh C, Friedrichs S, Levin M (2011). NM-Series of Representative Manufactured Nanomaterials, Zinc Oxide NM-110, NM-111, NM-112, NM-113, Characterisation and Test Item Preparation.

[41] Liu J, Hurt R H (2010). Environ Sci Technol.

[42] Liu J, Wang Z, Liu F D, Kane A B, Hurt R H (2012). ACS Nano.

[43] Loza K, Diendorf J, Sengstock C, Ruiz-Gonzalez L, Gonzalez-Calbet J M, Vallet-Regi M, Köller M, Epple M (2014). J Mater Chem B.

[44] Rehberg M, Krombach F, Pohl U, Dietzel S (2011). PLoS One.

[45] Liu T-M, Tai S-P, Yu C-H, Wen Y-C, Chu S-W, Chen L-J, Prasad M R, Lin K-J, Sun C-K (2006). Appl Phys Lett.

[46] Tai S-P, Wu Y, Shieh D-B, Chen L-J, Lin K-J, Yu C-H, Chu S-W, Chang C-H, Shi X-Y, Wen Y-C (2007). Adv Mater.

[47] Sauer U G, Vogel S, Aumann A, Hess A, Kolle S N, Ma-Hock L, Wohlleben W, Dammann M, Strauss V, Treumann S (2014). Toxicol Appl Pharmacol.

[48] Wang H S, Qiao X L, Chen J G, Ding S Y (2005). Colloids Surf, A.

[49] (2011). Standard operating procedures for characterisation of the selected manufactured nanomaterials types.

